# Personal experience with the remote check telehealth in cochlear implant users: from COVID-19 emergency to routine service

**DOI:** 10.1007/s00405-023-08045-2

**Published:** 2023-07-01

**Authors:** Marco Carner, Luca Bianconi, Gianfranco Fulco, Gennaro Confuorto, Davide Soloperto, Gabriele Molteni, Luca Sacchetto

**Affiliations:** Unit of Otolaryngology, Head and Neck Department, University of Verona, Piazzale L. A. Scuro, 10, 37100 Verona, Italy

**Keywords:** Cochlear implant, Remote check, Monitoring, Stable aided hearing

## Abstract

**Purpose:**

To critically illustrate the personal experience with using the “Remote Check” application which remotely monitors the hearing rehabilitation level of cochlear implant users at home and further allows clinicians to schedule in-clinic sessions according to the patients’ needs.

**Methods:**

12-month prospective study. Eighty adult cochlear implant users (females *n* = 37, males *n* = 43; age range 20–77 years) with ≥ 36 months of cochlear implant experience and ≥ 12 months of stable auditory and speech recognition level volunteered for this 12-month long prospective study. For each patient, at the beginning of the study during the in-clinic session to assess the stable aided hearing thresholds and the cochlear implant integrity and patient’s usage, the “Remote Check” assessment baseline values were obtained. “Remote Check” outcomes were collected at different times in the subsequent at-home sessions, to identify the patients that had to reach the Center. Chi-square test has been used for statistical analysis of the comparison of the “Remote Check” outcomes and in-clinic session results.

**Results:**

“Remote Check” application outcomes demonstrated minimal or no differences between all sessions. The at-home Remote Check application reached the same clinical outcomes as the in-clinic sessions in 79 out 80 of participants (99%) with high statistical significance (*p* < 0.05).

**Conclusions:**

“Remote Check” application supported hearing monitoring in cochlear implant users that were not able to attend the in-clinic review during COVID-19 pandemic time. This study demonstrates that the application can be a useful routine tool also for clinical follow-up of cochlear implant users with stable aided hearing.

**Supplementary Information:**

The online version contains supplementary material available at 10.1007/s00405-023-08045-2.

## Introduction

It is well known that 1-year post-activation most adult cochlear implant (CI) users have reached a stable plateau in hearing level and performance and require no or minimal clinical intervention at in-clinic follow-ups [1].

Due to the stability of the hearing level, distances needed to travel and growing overall costs, and CI users tend to cancel scheduled in-clinic follow-up appointments, which can negatively impact clinical daily routine. Moreover, the nation-wide lockdown, due to the COVID-19 pandemic, further worsened CI users’ compliance to attend annual appointments [2, 3].

To overcome these issues, such as the distances, and to ensure standard access to health care, some countries (e.g., UK, Australia, and France) have set up synchronous (i.e., real-time video-conferencing) and asynchronous (i.e., store and forward) telehealth services [4–7].

However, despite these technological advancements, the synchronous telehealth service relies on a strong Internet connection to support real-time communication [7].

“Remote Check” (RC) is the first CI asynchronous telehealth dedicated application available on smartphone and/or tablet (iOS or Android operating system) that uses wireless streaming via Bluetooth to allow aided hearing function follow-ups at home [8, 9]. Unlike the synchronous services, the RC tests can be conducted at any time when there is an adequate Internet connection.

Indeed, many studies [6, 9–11] have demonstrated that the tests used to monitor aided hearing level and performance within the at-home RC assessment are able to substitute the in-clinic assessments.

Therefore, this manuscript sought to present the results of one clinical pilot study on the use of RC assessment tool and its acceptance by CI users and clinicians in Italy. The results have been critically compared to previous similar international clinical investigations using RC application [6, 8, 9].

## Materials and methods

### Study design

This is a 12-month long prospective study. It includes non-randomized, repeated at home and in-clinic measurements on the same subject, where each CI user served as his/her own control. This study was approved by the Research Ethics Committee of Verona University Hospital and conformed to the standard set in the latest version of the Declaration of Helsinki (except for registration in a database). Verbal and written informed consent were obtained from all participants and/or parents/caregivers prior to the start of testing.

### Patients

Eighty adult CI users (females *n* = 37; males *n* = 43) with an age range of 20–77 years volunteered to participate in this study. The inclusion criteria for this study were: to be a CI user with a recent model of “Cochlear” CI series devices, compatible with the RC application; to have ≥ 36 months of CI experience; to have ≥ 12 months of a stable hearing level between 20 and 30 dBHL for 0.5–4 Hz and a stable speech recognition threshold (SRT) at 50–70%. Exclusion criteria were disability and/or medical conditions (e.g., visual impairment, neuro-developmental disorders, cognitive deficit, and patients who do not have support to take implant site photographs) that would restrict the use of the RC application.

### Remote check application description

The hearing assessments of the RC application are the Aided Threshold Test (ATT) for tonal threshold evaluation and the Digital Triplet Test (DTT) for the speech auditory performances. The application also includes: automated implant impedance test; data log activity for collection of usage data and sounds processor diagnostics; implant site photos; and a questionnaire.

Table 1 summarizes the issues investigated by the different RC tests: in situ surgical anomalies; electrode faults; tonal and speech hearing thresholds; sound quality; CI device integrity and patient’s usage. For each investigated issue, the cut-off values that indicate the need of an in-clinic assessment are also reported based on specific clinical experience and data from literature [8, 9] (Table 1).

**Table 1:** Issue investigated by RC tests at home and by the in-clinic tests during the annual check

Issue investigated	In-clinic test	Remote check test	Cut-off remote check values
In situ surgical anomalies	Visual inspection	Photos	Skin flap, soreness, etc.
Electrode faults	Impedance test	Impedance test	< 565 ohms (short circuit) > 30 kohms (open circuit)
Device usage	Datalog	Datalog	50% reduction daily use
Integrity device parts	Visual inspection	Questionnaire	Negative answers
Tonal hearing threshold	Tonal audiometry	Aided threshold test (ATT)	Threshold deterioration: > 5dbhl for all frequencies or > 35 dbhl for one frequency
Speech hearing threshold	Speech audiometry	Digital triplet test (Dtt)	> 2db snr modification (mean value)
Sound quality (poor)	Direct interview	Questionnaire	> 50% negative answers

The cut-off values (baseline modification) indicating the need of an in-clinic appointment are detailed

A detailed description of the different tasks is available elsewhere [6, 9], and hereby, only the main characteristics of the tests are briefly reported.

The hearing tests included in the RC applications (ATT and DTT) enable the clinician to evaluate the CI users’ auditory sensations. However, it must be noted that the results of these tests are not directly comparable to any in-clinic audiogram testing and were therefore compared with the baseline results obtained during the in-clinic first subject enrollment visit.

During ATT, CI users need to detect streamed pure tones across the speech frequencies to obtain a precise measurement of aided thresholds across the speech frequency spectrum. Frequencies are assessed in a specific order and 1 kHz tone is repeated. The test begins at 40 dB at each frequency with each step decreases to 1 dB close to threshold. For ATT, an average threshold deterioration ≥ 5 dBHL with respect to baseline values across all test frequencies or an aided threshold ≥ 35 dBHL at one or more frequencies were the criterions to determine the need for an in-clinic management of the patient.

The DTT is a self-test that uses monosyllabic digit triplets (i.e., 5–3–6) instead of sentences or monosyllabic words. The DTT is an adaptive test suitable for many CI users, including children, and allows to detect changes as a conventional speech in noise test [9, 10]. Results are reported as speech reception threshold (SRT) in dB signal-to-noise ratio (SNR). Two sets of eight triplets each are presented, and the score is the mean SNR of the 16 triplets. A modification ≥ 2 dB with respect to the baseline DTT values determined the need for a clinical control.

The automated impedance implant check allows for the detection of any changes to the implant electrodes and their effectiveness since the previous check. The software provides the range of impedances considered to be within normal limits [12]. Electrodes with impedances below 565 Ohms (short circuit) and above 30 kOhms (open circuit) should be deactivated during an in-presence management.

Furthermore, the RC assessment provides access to similar datalogging informations, accessible in the fitting software. RC datalog shows the usage data across time and daily since the last check. In contrast, the data displayed in-clinic sessions are aggregated view of the data since the last time the sound processor was connected to the fitting software. RC datalogs include time on air, time spent in use and in standby for accessories (TV streamer, mini mic, mobile devices, etc.) time spent in each program, time spent in scan scenes, proportion of time spent in various loudness scenarios, and time spent in forward focus.

The study participants were asked to photograph the implant areas under the coil, located behind the ear, and to photograph the outer ear canal, to allow for a remote review of the implant site (i.e., skin irritation/inflammation and flap/soreness).

CI users were also asked to complete a validate questionnaire [13, 14] for adults and children (Online Resource 1) that allows the patients to document detailed medical and health-related data, hearing performances, and device and/or training issues. The questionnaire includes the Speech, Spatial and Qualities of the Hearing Scale (SSQ) for adults or children. The SSQ was selected as it is a validated measure and scores that have been shown to stabilize after 12 months post-activation making it suitable for tracking performance changes over the longer term [13, 14].

The results obtained at-home are then transmitted via the cloud and are displayed on the web-based professional portal for the clinician to review and compare with the baseline outcomes. Once the clinician has addressed any concerns and completed the RC assessment review, he will then notify the CI user of the official results and any further recommendations, including, when necessary, an in-clinic appointment.

### Protocol of study

During a routine annually in-person control, to verify the stable aided hearing level of CI user and to document the good performances of the CI, confirming that no rehabilitative management was necessary, the first RC assessment (*T*_0_) was performed in the same session and completed during the appointment in clinic. This allowed to train and familiarize participants of the study to the correct use of the application and to establish a baseline to use as reference for the following RC at-home assessments.

Afterward, participants performed the RC assessments via smartphone application at home, every 4 months (*T*_1_, *T*_2_). These were scheduled by the clinician via a professional web-based portal and designed to fit the participant’s clinical needs. Tests were always administered in the same sequence.

At the end of every RC session, the clinician reviewed the results of each RC test to identify any relevant changes from baseline and to decide if an in-clinic follow-up assessment of the participant was required.

**Fig. 1:**
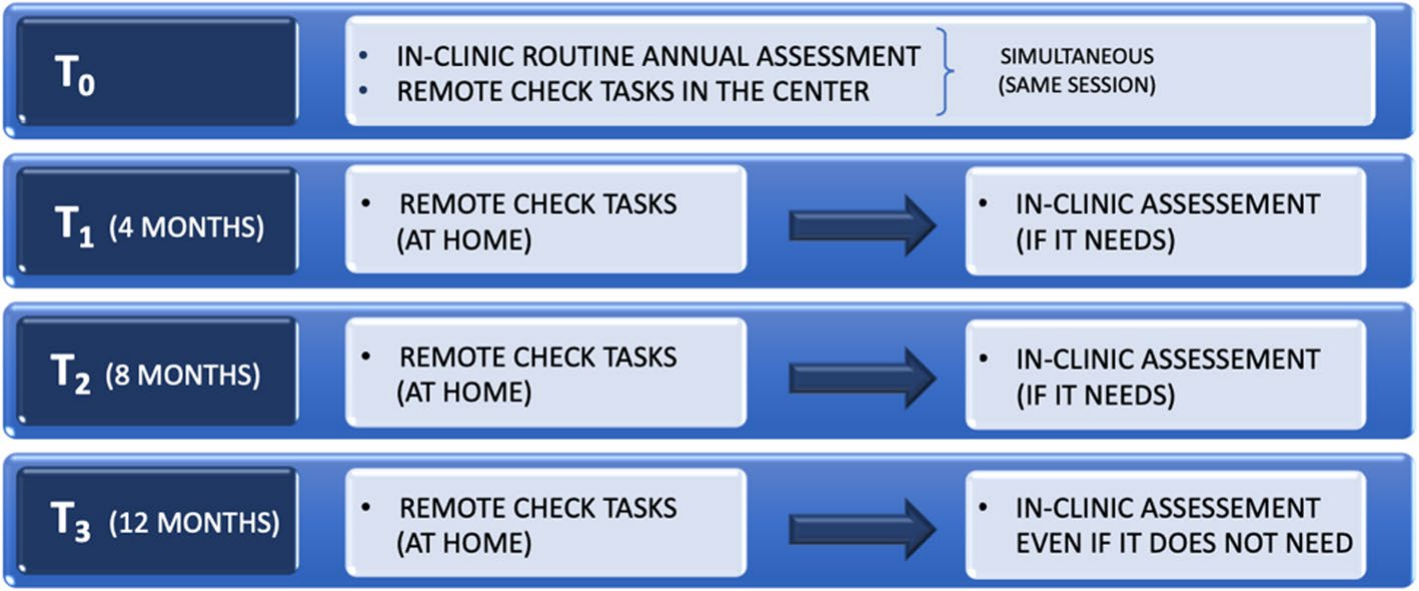
Protocol study schematic sequence

At the end of the study (*T*_3_) after the last RC assessment at home, an in-clinic evaluation was performed, even if not necessary (no abnormal issue), to confirm whether RC test battery had identified all the issues and that no clinical management was necessary (Fig. 1).

The review and evaluation of RC results were conducted by a fully licensed audiologist (M.C.) who has had > 30 years of experience in the field of hearing implant rehabilitation.

### Analyses and statistics

We used a Chi-square test for the statistical analysis to compare the at-home versus in-clinic results (number of CI users with abnormal issues successfully identified vs. number of patients were RC application failed to identify issues that required in-clinic assessment).

## Results

### RC application tests

The ATT was completed by all CI users. We found that the results performed at-home matched the baseline, obtained with the application during an in-clinic session, in all participants. The average aided hearing threshold for all frequencies was 25 dBHL (range 10–30 dBHL) and no CI user had a mean threshold modification ≥ 5dBHL, among the different RC sessions.

The DTT was completed by all participants with an average score of − 3.1 dB SNR (range − 7.8 to 2.1 dB SNR), along the entire time study. No or minimal difference (< 2 dB SNR) was observed among the sessions for all the patients.

Electrode impedances were within normal values in all 80 participants that used the direct RC impedance measurement. The electrode impedance values were confirmed in-clinic for 79 out of 80 patients (99%).

In one patient, RC datalog identified abnormal data (time use) not evidenced during the in-clinic evaluation.

Clear and useful photos were taken for all participants. No participants required any clinical action after both the RC assessment and direct clinician’s evaluation of the photos.

All participants completed the questionnaires during the RC assessments.

### Overall remote check assessment

In all but one participant of this study, the RC application outcomes were the same as in clinic assessment when determining whether the CI users required any further clinical action.

Chi-square analysis found that the number of CI users (79/80, 99%) where the test battery was successfully identified all issues recognized by the clinician during the in-presence session, was statistically significant (*p* < 0.05).

The RC application was easy or quick to use for 94% of the participants (*n* = 75), with an average duration of 28 min (range 20–43 min) vs. average duration of 92 min (range 45 min–3 h) for in-clinic assessments. To review the RC results took the clinician on average 15 min (range 12–20 min).

## Discussion

Lifelong rehabilitation and monitoring are provided to CI recipients via in-clinic assessments at specialized health centers. The assessments are scheduled annually, usually 1 year after the CI activation [15]. However, the majority of CI users reach a stable aided hearing level 1 year after the initial CI activation [1], leading to an overall reduction of in-clinic follow-up assessments. The reduction is due, in part, to the fact that most CI users no longer require any additional interventions during the follow-ups, i.e., no map or impedance change, no replacement for any device parts, and no surgical site issues (e.g., skin irritation) [1, 6, 9]. Howe and Mawman in 2015 [1] reported that over 89% of their study participants had stable if not better performance for aided tonal and speech audiometry at their annual in-clinic monitoring. Furthermore, 99 out of 100 participants had no change in their overall CI telemetry. Indeed, in our experience, 75% of CI users that attended an annual in-clinic review had stable aided hearing and did not require any further intervention.

In addition to the stability of the hearing level, CI users tend to either postpone or cancel scheduled in-clinic assessments, because of the distances needed to travel, overall costs, and more recently for the COVID-19 pandemic restrictions [1–3, 6, 7, 16]. With frequent cancelations perhaps negatively impacting clinical daily routine, new technological advancements, such as synchronous (i.e., real-time video-conferencing) and asynchronous (i.e., store and forward) telehealth services, have allowed CI users to undergo at-home clinical assessments [6, 7, 17]. RC has become the first CI telehealth asynchronous assessment tool that uses wireless streaming to enable comprehensive, easy, and reliable at-home self-testing of hearing function by the CI users, or their caregivers (person-centered CI care).

In this study on 80 patients implanted with a recent “Cochlear” device compatible with the application, the asynchronous RC assessment tool has been successful in defining whether CI users with stable aided hearing require any further clinical action. Furthermore, the RC assessment tool allows CI users to overcome the restrictive barrier of distance, while enabling clinicians the ability to save time for patients who have urgent needs.

A limitation of this study is that our experience has been conducted only with “Cochlear” implants. This application can be generalized in the future to include all types of CI, and very recently, RC tools have been proposed by other CI companies and they are being perfected and validated.

In this study, we used repeated-measures (at home and in-clinic for each CI user) design where each subject served as his/her own control group.

We found that only one (abnormal unrecognized electrode impedance values) out of the 80 participants were not identified by the RC application as needing an in-clinic review.

In general, the RC assessment tool identified all the problems usually recognized during the standard in-clinic review.

Moreover, we found that the overall RC assessments results were similar, if not better than the outcomes obtained in the previous investigations with the RC assessment tool [8, 9].

The ease and speed with which CI users can perform the RC assessment and the direct involvement of the clinician with fast and appropriate actions permitted the use of the RC application every 4 months, thus ensuring continuous monitoring of these users.

Most participants and parents/caregivers at the end of the study declared to prefer the self-managed RC application as a follow-up tool to determine aided hearing level and CI performance over in-clinic assessments.

In the literature, it is well known that self-management support interventions do not compromise overall health outcomes [18] and CI users that use self-management RC devices show a significant improvement in their auditory outcomes [6, 7, 9, 19, 20].

For clinicians, the RC assessment tool supports their clinical decision-making, thus allowing for the customization of care and prioritization of those who need additional support (complex cases). National health laws should be enacted to ensure equal digital technology access, privacy, security, and regulation of service payment to all RC users.

Previous studies have demonstrated the comparison of at-home RC test results versus in-clinic follow-up outcomes, showing a similar aided hearing level and CI performance between the two [5, 6, 9, 11]. Clearly, demonstrating the reliability and similarities, if not better results of at-home RC assessments when compared to the standard clinical care.

The positive experience reported in this study on RC application with 80 CI users clearly outlines that a careful process of decision-making about the patients suitable to use RC application is mandatory, because not all CI users should follow a telemedicine program, and RC tasks are not for everyone.

CI users with stable aided hearing should use RC application at home as a routine annual follow-up of their CI performances and hearing levels, instead of an in-clinic expensive and time-consuming check.

## Conclusion

RC application enables selected CI users with steady, correct CI functioning and with stable aided hearing to monitor their hearing performances (progress vs. worsening) at-home. Further, it helps clinicians to determine and plan for necessary clinical visits based on the patient needs. The RC application tool may perhaps play a critical future role in at-home CI performance assessments, considering the growing number of CI users and the decreasing number of specialized audiologists around the world. It is mandatory to accurately choose the patients that should use the RC application at home.

### Supplementary Information

Below is the link to the electronic supplementary material.

Online Resource 1 Remote Check Questionnaire: The RC questionnaire is designed to be self-administered by adult CI users or by parents of children with CI. It allows in detecting issues identified by clinicians during in-clinic session. (PDF 120 KB)

## Data Availability

All data generated during this study are contained in this published article.
