# Supplementary material for: Personal experience with the remote check telehealth in cochlear implant users: from COVID-19 emergency to routine service
Source: Eur Arch Otorhinolaryngol. 2023 Jul 1;280(12):5293–8. doi: 10.1007/s00405-023-08045-2 (PMC10620244; doi:10.1007/s00405-023-08045-2)
Supplement: Supplementary file 1 — Online Resource 1 Remote Check Questionnaire: The RC questionnaire is designed to be self-administered by adult CI users or by parents of children with CI. It allows in detecting issues identified by clinicians during in-clinic session. (PDF 120 KB) [file 405_2023_8045_MOESM1_ESM.pdf]

REMOTE CHECK QUESTIONNAIRE

MEDICAL ISSUES

1. When you touch the skin under the magnet, is there any numbness or pain?
    - ☐ No
    - ☐ Yes
2. Is the skin under the magnet red or discolored?
    - ☐ No
    - ☐ Yes
3. Is your sound processor causing soreness or any kind of discomfort?
    - ☐ No
    - ☐ Yes
4. Do you currently have an earache?
    - ☐ No
    - ☐ Yes
5. Is there any discharge from your ear?
    - ☐ No
    - ☐ Yes

FITTING ISSUES

6. Have you changed the way you use the hearing aid on your other ear? e.g. Have you stopped using a hearing aid or received a new one?
    - ☐ No
    - ☐ Yes
    - ☐ I don't wear a hearing aid

    If Yes, in what way? ____
7. Since your last clinic appointment, have you experienced any of the following while using your sound processor? (Tick all that apply).
    - ☐ Painful sensations
    - ☐ Twitching near the eye or anywhere on the face
    - ☐ Sounds that are uncomfortably loud
    - ☐ Sounds that feel too soft or not loud enough
    - ☐ Interruption of sound
    - ☐ Sounds that feel louder in one ear than the other
    - ☐ None of the above

SOUND QUALITY ISSUES

8. Do you think your hearing has changed since your last clinic appointment or Remote Check?
    - ☐ No
    - ☐ Yes

    If Yes, in what way? ____
9. Does someone you know (e.g., a family member, friend, etc.) think that your hearing has become worse?
    - ☐ No
    - ☐ Yes

THE NEXT SET OF QUESTIONS WILL ASK YOU TO THINK ABOUT THE LAST TIME YOU WERE IN CERTAIN SCENARIOS (INDICATE A NUMBER FROM 0 -NOT AT ALL- TO 10 -PERFECTLY-)

10. You are talking with one other person and there is a TV on in the same room. Without turning the TV down, can you follow what the person you're talking to says? ___
11. You are listening to someone talking to you, while at the same time trying to follow the news on TV. Can you follow what both people are saying? ___
12. You are in conversation with one person in a room where there are many other people talking. Can you follow what the person you are talking to is saying? ___
13. You are in a group of about five people in a busy restaurant. You can see everyone else in the group. Can you follow the conversation? ___
14. You are with a group and the conversation switches from one person to another. Can you easily follow the conversation without missing the start of what each new speaker is saying? ___
15. You are outside. A dog bark loudly. Can you tell immediately where it is, without having to look? ___
16. Can you tell how far away a bus or a truck is, from the sound? ___
17. Can you tell from the sound whether a bus or truck is coming towards you or going away? ___
18. When you hear more than one sound at a time, do you have the impression that it seems like a single jumbled sound? ___
19. When you listen to music, can you make out which instruments are playing? ___
20. Do sounds that you can hear easily seem clear to you (not blurred)? ___
21. Do you have to concentrate very much when listening to someone/something? ___

DEVICE'S CHECK ISSUES

22. If your processor falls off often, would you like help keeping it in place?
    - ☐ No, I am managing well
    - ☐ Yes, I would like help
    - ☐ My processor does not fall off too often
23. Do you have any concerns about how well your implant/sound processor is working?
    - ☐ No
    - ☐ Yes

    If Yes, what concerns do you have? ______________________________
24. When did you last change your microphone cover?
    - ☐ In the past 3 months
    - ☐ More than 3 months ago
    - ☐ I can't remember
25. How often do you use the dry aid kit?
    - ☐ Every day
    - ☐ Not every day
    - ☐ Not sure
26. Has anything happened since your last clinic appointment or Remote Check that might have affected how well your implant/sound processor is working? (e.g., Was your processor dropped in a pool? Did you bump your head on the implant site? Etc.)
    - ☐ No
    - ☐ Yes

    If Yes, please specify what happened. ______________________________
27. Do you need help getting spare parts for your processor? (e.g., microphone covers, retention accessories, batteries, Etc.)
    - ☐ No
    - ☐ Yes

    If Yes, please specify what you would like help with. ______________________________

LISTENING ACTIVITIES ISSUES

28. Has your participation in any of the following activities decreased since your last appointment? (Tick all that apply).
    - □ I watch TV less often
    - □ I talk in a group less often
    - □ I have been having fewer conversations in noisy places
    - □ I don't listen to music as much
    - □ I don't talk on the phone as much
    - □ I don’t attend church/lectures as much
    - □ I talk to children less
    - □ I don't have as many one-on-one conversations
    - □ I am less likely to talk to people I don’t know well
    - □ None of the above (My participation in the above situations has remained the same or increased since my last appointment.)
29. Do you need training on any of the following activities? (Tick all that apply).
    - □ Changing zinc air/rechargeable batteries
    - □ Changing the volume on your sound processor
    - □ Changing programs on your sound processor
    - □ Using the dry aid kit or Home Charger
    - □ Changing microphone covers
    - □ Using tele-coil
    - □ Using wireless accessories
    - □ Understanding lights and beeps on your sound processor
    - □ None of the above (I don’t need training on any of the above)
30. Would you like to discuss any other issues with your clinician?
    - □ No
    - □ Yes

    If Yes, please specify what you would like to discuss. ____

For pediatric CI users, the same questions, in a simplified and customized version, are administered by their parents.
